# Shepherd: accurate clustering for correcting DNA barcode errors

**DOI:** 10.1093/bioinformatics/btac395

**Published:** 2022-06-16

**Authors:** Nik Tavakolian, João Guilherme Frazão, Devin Bendixsen, Rike Stelkens, Chun-Biu Li

**Affiliations:** Department of Mathematics, Stockholm University, Stockholm 10691, Sweden; Department of Zoology, Stockholm University, Stockholm 10691, Sweden; Department of Zoology, Stockholm University, Stockholm 10691, Sweden; Department of Zoology, Stockholm University, Stockholm 10691, Sweden; Department of Mathematics, Stockholm University, Stockholm 10691, Sweden

## Abstract

**Motivation:**

DNA barcodes are short, random nucleotide sequences introduced into cell populations to track the relative counts of hundreds of thousands of individual lineages over time. Lineage tracking is widely applied, e.g. to understand evolutionary dynamics in microbial populations and the progression of breast cancer in humans. Barcode sequences are unknown upon insertion and must be identified using next-generation sequencing technology, which is error prone. In this study, we frame the barcode error correction task as a clustering problem with the aim to identify true barcode sequences from noisy sequencing data. We present Shepherd, a novel clustering method that is based on an indexing system of barcode sequences using *k*-mers, and a Bayesian statistical test incorporating a substitution error rate to distinguish true from error sequences.

**Results:**

When benchmarking with synthetic data, Shepherd provides barcode count estimates that are significantly more accurate than state-of-the-art methods, producing 10–150 times fewer spurious lineages. For empirical data, Shepherd produces results that are consistent with the improvements seen on synthetic data. These improvements enable higher resolution lineage tracking and more accurate estimates of biologically relevant quantities, e.g. the detection of small effect mutations.

**Availability and implementation:**

A Python implementation of Shepherd is freely available at: https://www.github.com/Nik-Tavakolian/Shepherd.

**Supplementary information:**

Supplementary data are available at *Bioinformatics* online.

## 1 Introduction

DNA barcodes are short DNA sequences that are introduced into a cell population to identify individuals and their offspring. These barcodes are passed on from generation to generation and can be used to track the relative counts of lineages over time (Blundell and Levy, 2014; Masuyama *et al.*, 2019; Nguyen Ba *et al.*, 2019; Weinreb and Klein, 2020). This technology is useful for analyzing the evolutionary dynamics of a population. For example, it has been used to infer the effects of mutations in populations of *Saccharomyces cerevisiae* (Johnson et al., 2019; Levy *et al.*, 2015) and to track the progression of breast cancer in humans (Nguyen *et al.*, 2015).

In general, the barcodes are unknown random DNA sequences. Once established in a population, the barcodes are identified through sequencing by synthesis, a process which involves generating millions of copies of each barcode using polymerase chain reaction (PCR) amplification. This is followed by a sequencing step, whereby each barcode sequence is identified. The counts of the identified sequences estimate the relative counts of the barcodes in the population. However, this ignores the fact that the sequencing process is error prone and assumes that each identified sequence corresponds to a unique barcode in the population. Both PCR amplification and sequencing can introduce errors in the identification of the barcodes, typically in the form of substitution errors, through which one or more nucleotides in a barcode are misidentified as different nucleotides. The substitution error rate for the Illumina sequencing platform (Bentley *et al.*, 2008) was estimated by Pfeiffer *et al.* (2018) to be ∼0.24% per base and ∼6.4% of the sequences were found to contain at least one substitution error. To correctly determine the relative counts of the barcodes, these errors must be identified and corrected.

We first define an *error sequence* as a sequence that originated through one or more substitution errors in the identification of a barcode. The original barcode is the *source barcode* of the error sequence. The error correction task can then be viewed as a clustering problem that groups similar sequences together: All error sequences that have the same source barcode should belong to the same cluster, together with their source barcode.

The main challenge of this task is that the number of unique sequences can be in the millions and the number of barcodes can be in the hundreds of thousands. Since clustering involves grouping similar items, a standard approach is to compute all pairwise distances between the items before applying some clustering algorithm (Frey and Dueck, 2007). However, this approach is extremely computationally costly with millions of unique sequences. In addition, the number of barcodes is unknown beforehand, making the task more difficult since the cluster counts cannot be used as a guide to find accurate clusters.

Bartender (Zhao *et al.*, 2018) and Starcode (Zorita *et al.*, 2015) are examples of recent methods for clustering barcode reads. These methods avoid fully computing all pairwise distances by utilizing various prioritization schemes. Nevertheless, they do not incorporate explicit estimates of the error rates associated with PCR amplification and sequencing. Explicit error rate estimates enable approximation of the probability distribution of error sequences. Knowledge of this distribution allows for more accurate classification of a sequence as either a true barcode or an error sequence.

Here we present Shepherd, a new method for error correction of barcode reads. Shepherd is based on the idea of partitioning the barcode sequences into non-overlapping *k*-mers that are substrings of length *k*. These substrings are then used to construct an indexing system termed the *k*-mer Index, similar to a book indexing system in a library, which allows us to efficiently find the local neighborhood for a given sequence. This local neighborhood includes only sequences that are within a predefined distance to the sequence under consideration, without having to compute its distance to all other sequences. Furthermore, Shepherd employs a Bayesian hypothesis test that explicitly incorporates the substitution error rate to discriminate true barcodes from error sequences.

The main achievement of Shepherd is that it offers a substantial improvement in error correction accuracy over other state-of-the-art methods. Specifically, Shepherd offers significant improvements for tracking low count lineages accurately. When compared with existing methods, Shepherd achieves 10 to 150 times fewer spurious lineages on synthetic single and multiple time point data. Furthermore, Shepherd provides highly accurate and unbiased barcode count estimates throughout the count range. On the experimental Illumina HiSeq data (Levy *et al.*, 2015), we obtain results that are consistent with the improvements in the synthetic data benchmark.

In Section 2, we provide a detailed description of the *k*-mer indexing system that enables efficient identification of sequence neighborhoods, and describe the clustering procedure that uses this *k*-mer indexing system to reliably correct substitution errors. In Section 3.1, we evaluate the error correction accuracy of Shepherd on synthetic data for both a single time point and multiple time points. In Section 3.2, cluster validation measures are used to evaluate the error correction accuracy of Shepherd on experimental Illumina HiSeq data (Levy *et al.*, 2015), with comparisons to the current state-of-the-art methods, Bartender and Starcode. We discuss the results and the significance of our new approach in Section 4.

## 2 Materials and methods

In this section, we will explain each step performed by Shepherd to find accurate barcode clusters. In Section 2.1, we introduce the *k*-mer indexing system which forms the backbone of the method and enables efficient identification of neighbors for each sequence. In Section 2.2, the main clustering procedure is described.

### 2.1 Finding sequence neighborhoods: the *k*-mer Index

The purpose of the *k*-mer indexing system is to enable computationally inexpensive identification of all sequences that are similar to a given sequence. Before the *k*-mer Index can be constructed, a sensible distance metric between the sequences must be defined to specify what constitutes similarity. With error sequences arising from substitution errors, it is natural to use the Hamming distance as our distance metric. Let *S_a_* and *S_b_* denote two sequences with the same length *l*, the Hamming distance between the sequences is the number of nucleotide substitutions needed to convert one sequence to the other,

(1) $$h(S_{a},S_{b})=\sum_{j=1}^{l}I(S_{a}[j]\neq S_{b}[j]),$$

where S[j] is the nucleotide at the *j*th position of the sequence *S* and *I* denotes the indicator function defined by,

(2) $$I(x\neq y)=\{\begin{array}{c} 1\;\;\text{if}\;\;x\neq y,\\ 0\;\;\text{if}\;\;x=y. \end{array}$$

We next define the *ϵ*-neighborhood of a given sequence as the set of all sequences with Hamming distance smaller than or equal to *ϵ* from it. We want to choose *ϵ* so that all error sequences of a barcode are within its *ϵ*-neighborhood. However, we do not want *ϵ* to be larger than necessary since the time and memory complexity of the method increases with larger *ϵ*. Therefore, a suitable value for *ϵ* offers a trade-off between accuracy and performance. For now, we assume that an appropriate value for *ϵ* has been chosen and detail the procedure for determining *ϵ* in Supplementary Section S3B. Our goal is to efficiently find the *ϵ*-neighborhood of each unique sequence.

To this end, consider each sequence as a set of non-overlapping *k*-mers that are substrings of length *k*. Let *l* denote the barcode length, each choice of *k* partitions the sequence into a set of p k-mers. One has p=l/k if *l* is a multiple of *k*. Otherwise, the integer quotient defines the number of partitions with length *k* and there is one additional partition of length *r* with *r* the remainder.

**Fig. 1.**
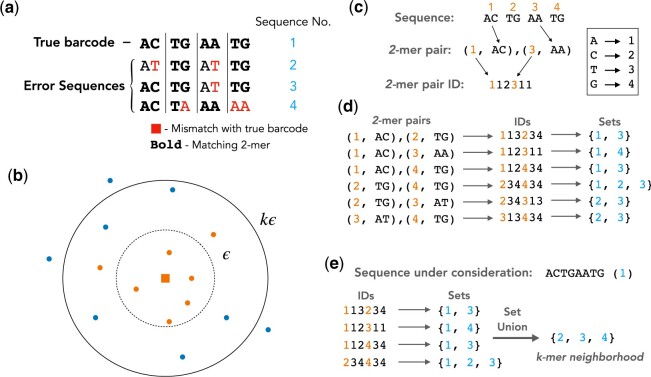
(**a**) Illustration of the pigeonhole principle for *l *=* *8 and *k *=* *2 (i.e. p=l/k=4). Sequences 2 and 3 are within Hamming distance 2 of the true barcode. It follows from the pigeonhole principle that these error sequences share two or more 2-mers with the true barcode. Since Sequence 4 has Hamming distance 3 to the true barcode the pigeonhole principle only guarantees that it shares one 2-mer with it. Nevertheless, Sequence 4 still shares two 2-mers with the true barcode since two of its errors appear in the same 2-mer. (**b**) A given sequence (orange square) surrounded by its neighbors (dots) in sequence space. The orange dots are the *k*-mer neighbors of the given sequence, i.e. all sequences that share at least p−ϵ k-mers with it. The blue dots are sequences not included in the *k*-mer neighborhood. The dashed circle is the *ϵ*-neighborhood of the sequence and the solid circle is the boundary for the *k*-mer neighbors, i.e. no *k*-mer neighbor appears outside the solid circle. Note that *l* is a multiple of *k* in this case and that all *ϵ*-neighbors of the sequence are also *k*-mer neighbors, this is guaranteed by the pigeonhole principle. (**c**) Illustration of how a pair of 2-mers are converted into a combination ID. First a pair of 2-mers is selected. Each 2-mer has a location in the sequence specified by the orange numbers. The 2-mer pair is then converted to an ID by assigning each of its nucleotides to a number specified by the conversion table on the right. (**d**) The *k*-mer Index for the set of sequences from the panel a, including only the 2-mer pairs shared by at least two sequences in the dataset. The blue numbers correspond to the sequence numbers specified in the panel a. Furthermore, the *k*-mer Index only includes the combination IDs with the corresponding sets and the 2-mer pairs (leftmost column) are only included here for illustrative purposes. (**e**) A schematic showing the process of finding the *k*-mer neighborhood of Sequence 1 from the panel a using the *k*-mer Index from the panel d. First all combination IDs of Sequence 1 are found and the corresponding sets are obtained from the *k*-mer Index. The set union of the sets yields the set of all sequences that share at least one combination ID with Sequence 1. By excluding Sequence 1 from this set we obtain its *k*-mer neighborhood (A color version of this figure appears in the online version of this article.)

If a sequence is within distance *ϵ* of another sequence, the number of *k*-mers shared by the two sequences is at least p−ϵ. This follows from the pigeonhole principle exemplified in Figure 1a. The figure shows an example with Sequences 2 and 3 within a distance *ϵ *= 2 from the true barcode. Consequently, these error sequences share two or more 2-mers with the true barcode. This is because we have at most two errors that occur in four 2-mers. Placing one error in each 2-mer minimizes the number of matching 2-mers, but always leaves two 2-mers error free. In general, the pigeonhole principle guarantees that the set of sequences sharing p−ϵ or more *k*-mers with a given sequence includes all sequences in the *ϵ*-neighborhood of the sequence. We call this set the *k*-mer neighborhood of the given sequence and require that *k* is chosen such that p−ϵ>0, i.e. if two sequences have Hamming distance *ϵ*, we require that they share at least one *k*-mer.

Note that sequences outside of the *ϵ*-neighborhood might also be included in the *k*-mer neighborhood. For example, this is the case for Sequence 4 in Figure 1a. This sequence appears in the 2-mer neighborhood of the true barcode for *ϵ *= 2, despite having Hamming distance 3 to the true barcode. Nevertheless, since at least p−ϵ k-mers match, the *k*-mer neighborhood is bounded by a certain Hamming distance threshold. When *l* is a multiple of *k*, this threshold is given by kϵ. (Given that two sequences share exactly p−ϵ k-mers, they have *ϵ* distinct *k*-mers when *l* is a multiple of *k*. The maximum Hamming distance between them corresponds to the case when every position in the distinct *k*-mers is a mismatch.) An illustration of the sequence neighborhoods is shown in Figure 1b.

In general, there can be several values for the substring length *k* satisfying the constraint p−ϵ>0. There are two primary considerations when choosing *k*. Firstly, *k* should not be too large such that the *k*-mer neighborhood of a sequence contains many more sequences than its *ϵ*-neighborhood, as a large *k*-mer neighborhood increases the search time for finding the *ϵ*-neighbors. Secondly, *k* should not be too small since small *k* increases the size of the *k*-mer Index defined below by increasing the number of *k*-mer combinations. In Supplementary Section S3C, we provide details on how *k* is automatically determined to optimize the computational cost of finding the *ϵ*-neighborhoods.

For fixed *p* and *ϵ* there are ${}^{p}C_{p-\epsilon }$ ways for two sequences to share p−ϵ k-mers, where ${}^{m}C_{n}$ denotes the number of possible combinations when choosing *n* items from a total of *m* items. Equivalently, there are ${}^{p}C_{p-\epsilon }$ ways to choose p−ϵ k-mers from a sequence. Each one of these combinations is called a *k*-mer combination.

The *k*-mer Index is a lookup table containing a collection of ID-set pairs: the ID in the pair is a *k*-mer combination identification number, and the corresponding set in the pair is the set of all unique sequences that contain that *k*-mer combination. Figure 1d shows the *k*-mer Index for the sequences in Figure 1a, excluding the *k*-mer combinations that only appear in a single sequence. To construct the *k*-mer Index, the following steps are performed for each sequence in the dataset:

1. Find the ${}^{p}C_{p-\epsilon }$ combinations of *k*-mers for the sequence under consideration. For example, if the barcode length is *l* = 8 and the number of partitions is *p* = 4 as in Figure 1a, the number of 2-mer pairs is ${}^{4}C_{2}=6$ for *ϵ* = 2.
2. Convert each of these combinations into a combination ID, a number that uniquely represents the *k*-mer combination. This procedure is illustrated in Figure 1c for the case when *l* = 8 and *p* = 4.
3. Is the combination ID an established ID in the *k*-mer Index? **No:** Add the combination ID to the Index, the corresponding set is the set containing only the sequence under consideration. **Yes:** Query the index using the combination ID to find the set of sequences from previous iterations that share the *k*-mer combination. Then add the current sequence to that set, updating the set in the Index. Figure 1d shows the final sets that arise from this procedure for the sequences in Figure 1a.

The *k*-mer Index is constructed once the above steps are performed for each sequence. Given a sequence, the index can be used to find its *k*-mer neighborhood. We query the index with the combination IDs of each sequence to find all sequences that share at least one combination with the given sequence. These sequences constitute its *k*-mer neighborhood. Figure 1e shows the process of finding the *k*-mer neighborhood of the true barcode from Figure 1a, using the *k*-mer Index from Figure 1d. Once the *k*-mer neighborhood of a sequence is constructed, its *ϵ*-neighborhood can simply be found by keeping the sequences in the *k*-mer neighborhood that are within Hamming distance *ϵ*.

We note that the most direct way of finding *ϵ*-neighborhoods is computing all pairwise Hamming distances between the sequences. However, the method presented here based on the *k*-mer Index is significantly more efficient. This is because the construction of the *k*-mer Index only requires one pass over the unique sequences. In contrast, the computation of all pairwise Hamming distances requires $O(N^{2})$ of iterations, with *N* the number of unique sequences in the dataset. Once constructed, the index enables us to efficiently find the *k*-mer neighborhoods, narrowing the search for the *ϵ*-neighbors considerably.

### 2.2 Clustering using *k*-mer neighborhoods

In this section, we will describe the simple clustering procedure used to identify true barcodes and to group them with the error sequences that originated from them. The procedure is based on the observation that sequences with very high counts are unambiguously true barcodes, whereas sequences with low counts may be error sequences. We assume that the *k*-mer Index has already been constructed for some distance threshold *ϵ* and substring length *k*. Sequences classified as true barcodes by Shepherd are referred to as putative barcodes since they could still be error sequences due to classification errors.

In the first step of the clustering procedure, we iterate over the unique sequences one by one in descending order of read counts and use the *k*-mer Index to find their *k*-mer neighborhoods. The first sequence with the highest read count is always classified as a putative barcode. Subsequent sequences are also classified as putative barcodes as long as none of their *ϵ*-neighbors are putative barcodes. Indeed, if a sequence is an error sequence, one would expect that its source barcode is a nearby putative barcode with a higher read count.

However, if a putative barcode is found in the *ϵ*-neighborhood of the sequence under consideration, we cannot conclude if the sequence is an error sequence or a true barcode close to a putative barcode. Consequently, the naive strategy of classifying a sequence as an error sequence whenever it has a putative barcode as an *ϵ*-neighbor can lead to misclassification. The risk of misclassification is higher when the barcode length is short and *ϵ* is large, since these factors increase the likelihood that true barcodes are within distance *ϵ* of each other.

To avoid erroneous classification of sequences with nearby putative barcodes, a Bayesian statistical test is used to determine if the sequence under consideration is either a true barcode or an error sequence originating from its closest putative barcode. If the sequence is classified as an error sequence, it is clustered with its closest putative barcode. In the rare cases when two or more putative barcodes have the same Hamming distance to the sequence under consideration, the one with the higher read count is considered. We refer to Supplementary Algorithm S1 for a pseudo-code description of the procedure.

Our clustering procedure performs a statistical test every time a putative barcode is in the *ϵ*-neighborhood of the sequence under consideration. To reduce computational time, this test can be omitted for cases that are unambiguous, e.g. when the sequence has a very high read count it is almost certainly a true barcode. Furthermore, a truncated Hamming distance can be used to reduce computational time. We refer to Supplementary Section S3D for a detailed description of how the clustering procedure is optimized.

#### 2.2.1 Correcting insertion and deletion errors

In general, the rate of Indel sequencing errors is significantly lower than the rate of substitution errors. However, for sequences with long homopolymers it has been shown that the Indel error rate can be significantly higher (Minoche, *et al.*, 2011). In these sequences, single insertion or deletion errors may occur often enough that they must be corrected for to ensure accurate count estimation.

Shepherd corrects single insertion and deletion errors using a post-processing procedure. First, all sequences of length *l *+* *1 with single insertion errors are processed. For each of these sequences, we consider the *l *+* *1 possible single deletions that yield a sequence of the correct length *l*. If one of these sequences is a putative barcode identified in the main clustering procedure, we assign the sequence to that putative barcode. Analogously, we correct single deletion errors by processing all sequences of length *l* − 1 and considering the *l* possible single insertions that yield a sequence of the correct length.

#### 2.2.2 Bayesian statistical test

When a given sequence is within a Hamming distance *ϵ* from a putative barcode, it can either be an error sequence or a true barcode. Shepherd uses a statistical test to decide between the two cases (or hypotheses). The test accounts for the sequence under consideration and its read count, together with the sequence of the closest putative barcode and its read count. An error rate per nucleotide *ρ*, which is the estimated probability that a substitution error occurs at a nucleotide in a barcode, is also used to determine the likelihood of each hypothesis. We assume that the combined error rates of PCR amplification and sequencing are the same at each nucleotide position.

Let *S_c_* and *f_c_* denote the sequence under consideration and its read count, respectively. Furthermore, let *S_p_* denote the sequence of the neighboring putative barcode with read count *f_p_*. The Hamming distance between the sequences is given by *d*, such that d≤ϵ.

We will consider two competing models for the sequence under consideration. In the first model, *M*_1_, the sequence *S_c_* is an error sequence that originated from the nearby putative barcode *S_p_* through substitution errors. In the second model, *M*_2_, the sequence *S_c_* is a true barcode. The likelihood of each model, $P(S_{c},f_{c}|S_{p},f_{p},\rho ,M_{i})$ with *i *=* *1 or 2, is the probability of observing the sequence *S_c_* with read count *f_c_* given each model. The ratio of the likelihoods for each model is known as the Bayes Factor for decision-making. An expression for each likelihood can be found (see details in Supplementary Section S1) allowing us to obtain the following computable form for the logarithm of the Bayes factor *K*,

(3) $$\begin{array}{c} \ln K=\ln\frac{P(S_{c},f_{c}|S_{p},f_{p},M_{1})}{P(S_{c},f_{c}|S_{p},f_{p},M_{2})}\\ =\ln{(}^{\hat{n}}C_{f_{c}}{\hat{p}}_{\text{pc}}^{f_{c}}{\left(1-{\hat{p}}_{\text{pc}}\right)}^{\hat{n}-f_{c}})+\ln{\hat{p}}_{\text{pc}}+l\ln4+\ln f_{\text{max}}. \end{array}$$

Here $\hat{n}$ is an estimate of the true count of the putative barcode *S_p_* and $f_{\text{max}}$ is the highest read count observed in the data. The probability of converting the sequence *S_p_* to *S_c_* by substitution errors is estimated by ${\hat{p}}_{\text{pc}}={\left(\rho /3\right)}^{d}{\left(1-\rho \right)}^{l-d}$ (see Supplementary Section S1A). This estimate is based on the assumption that once a substitution error occurs, all three nucleotides that could replace the original one are equally likely to be picked.

To decide if the sequence *S_c_* is a true barcode or an error sequence, a threshold for the log-Bayes factor (Eq. 3) must be supplied. In this study, we choose the threshold equal to –4 as suggested by Kass and Raftery (1995) and refer to Supplementary Section S1 for details regarding the Bayesian statistical test.

### 2.3 Multiple time point error correction with Shepherd

So far we have covered error correction of barcode reads from a single time point. However, for the purpose of lineage tracking, barcode reads from multiple time points have to be processed. Specifically, error correction must be performed at each time point and the barcodes from one time point should be connected to the corresponding barcodes from different time points. Furthermore, some barcodes may not be identified in the first time point due to low sequencing coverage, but their counts may rise in later time points. Shepherd has the novel capability of identifying emerging barcodes at later time points. This enables more accurate lineage tracking, especially when the sequencing coverage is low.

Shepherd only performs clustering for the first time point using the procedure described in Section 2.2. For subsequent time points, Shepherd treats the error correction task as a classification problem and assigns each sequence to its closest putative barcode within distance *ϵ* from the previous time point. If no putative barcode is within distance *ϵ* of the sequence, it is added to the set of unassigned sequences and processed separately at a later stage. If two or more putative barcodes have the same Hamming distance to the sequence under consideration and appear in its *ϵ*-neighborhood, the sequence is assigned to the one in the higher count cluster.

Barcodes emerging at later time points may appear within the *ϵ*-neighborhoods of existing putative barcodes. To resolve these cases, Shepherd uses the statistical test from Section 2.2.2 to separate emerging barcodes from existing ones. This procedure has the additional benefit of correcting false negatives introduced in the first time point. Specifically, if two true barcodes are merged in the first time point, they will be separated at a later time point if their count discrepancy increases. We refer to Supplementary Section S2 for a detailed description of the error correction procedures for multiple time points.

## 3 Results

In this section, we evaluate the performance of Shepherd and compare it with other state-of-the-art methods for error correction of DNA barcodes, namely, Bartender and Starcode (Zhao *et al.*, 2018; Zorita *et al.*, 2015). In Section 3.1, the methods are compared on synthetic data, for which the true cluster labels, i.e. true sequences, are known. In Section 3.2, we compare the methods using the lineage tracking data of *S.cerevisiae* obtained by Levy *et al.* (2015) and perform clustering validation to assess the results.

### 3.1 Synthetic data

The evaluation of the methods for a single time point are based on three synthetic datasets. The synthetic barcodes have 20 random nucleotides and 6 constant nucleotides. This configuration was chosen to imitate barcodes produced in actual sequencing-based lineage tracking systems (Levy *et al.*, 2015).

Once the barcode sequences are generated, each one is assigned a count by drawing a sample from the exponential distribution with mean 100, and setting the count to the least integer greater than or equal to the sample (the ceiling function). To simulate substitution errors, we assume a constant per nucleotide error rate. Detailed procedures for generating the synthetic datasets are provided in Supplementary Section S4A.

The synthetic datasets are summarized in Table 1. The error rate for Dataset A is chosen to be close to the error rate estimated experimentally by Pfeiffer *et al.* (2018) for the Illumina sequencing platform. The error rates in Datasets B and C are set to be higher to account for the error rate differences between sequencing platforms, and to test the performance of the different error correction methods in large error conditions. All three methods, Shepherd, Bartender and Starcode, were tested using their default settings when possible, to yield results a user would obtain without re-running the algorithms to adjust the parameters.

**Table 1.** Summary of synthetic datasets

Dataset	A	B	C
Unique sequence count	3 428 741	5 773 822	2 591 318
True barcode count	499 656	499 320	99 507
Error rate	0.33%	0.66%	2%

*Note*: All datasets have barcodes with a total barcode length of 26, with 20 random nucleotides and 6 constant nucleotides.

For Shepherd, the parameter *ρ* was estimated from the data using the procedure described in Supplementary Section S3A. For all datasets, the relative difference between the true error rate and the estimated error rate was <1.5%. The threshold for log-Bayes factor was set to its default value –4. This threshold implies that the likelihood of model *M*_2_ must be at least around 55 times larger than the likelihood of model *M*_1_ for the sequence to be classified as a true barcode.

The motivation for favoring model *M*_1_ is that error sequences classified as true barcodes are generally more disruptive than true barcodes classified as error sequences for the purpose of lineage tracking. This is because in the former case an entirely new spurious lineage is created, whereas the latter case involves a relatively mild distortion of the estimated barcode counts. The other parameters of Shepherd are summarized in Supplementary Table S1 and were automatically determined using the procedures described in Supplementary Section S3. The parameters of Bartender are set to match the ones used by Zhao *et al.* (2018) on similar data and Starcode is applied with its default settings and the distance threshold set to 2.

To compare the clustering results of the methods, we consider the false positive count (FPC) and false negative count (FNC) on each dataset. A false positive is an identified cluster that does not correspond to a true barcode. A false negative is a true barcode that is clustered together with another true barcode with higher count. In other words, a false positive is a spurious lineage and a false negative is a true barcode that is incorrectly classified as an error sequence. A higher FPC implies a large number of spurious low count lineages, since most false positives are small groups of error sequences from a common source barcode. Furthermore, a high FNC indicates that the method is under-clustering by merging different barcodes. Table 2 shows the FPC and FNC for each method on each synthetic dataset. While the FNC is similar for all methods, the FPC is substantially lower for Shepherd across all datasets. This difference in FPC increases with the error rates of the datasets, suggesting that Shepherd performs better in large error conditions.

**Table 2.** The false positive count (FPC) and false negative count (FNC) for each method on each synthetic dataset

Dataset	A		B		C	
Measure	FPC	FNC	FPC	FNC	FPC	FNC
Shepherd	45	66	482	82	461	50
Bartender	1979	47	14 100	62	59 554	6
Starcode	7045	91	26 289	99	78 956	4

**Fig. 2.**
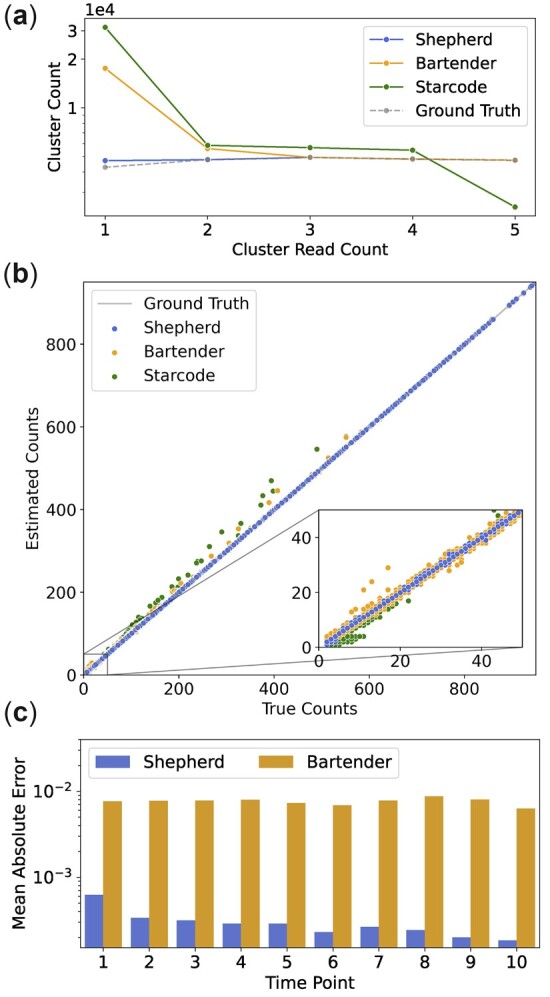
(**a**) The number of clusters with low read counts (<6) for each method compared to the ground truth on Dataset B. (**b**) Estimated barcode counts compared to the true counts for each method on Dataset B. Only true barcodes identified by all three methods are displayed. True barcodes for which all three methods estimated the same count are excluded to emphasize differences in the estimated counts. (**c**) The mean absolute difference between the true barcode counts and the estimated counts of Shepherd and Bartender at each time point. For each time point, only true barcodes identified by both methods are included in the comparison

Figure 2a shows the count distribution of the low count putative barcodes identified by each method on Dataset B compared to the ground truth. For higher count putative barcodes, the cluster read counts of all methods closely match the ground truth. However, as a consequence of the high FPCs of Bartender and Starcode when compared with Shepherd (see Table 2), these methods overestimate the number of clusters with low read counts, as can be seen from Figure 2a. In particular, the number of single read clusters is estimated to be around four times higher for Bartender and seven times higher for Starcode, when compared with the ground truth. The corresponding plots for Datasets A and C are given in Supplementary Figures S1 and S2, respectively.

The FPC and FNC provide information about the ability of each method to distinguish true barcodes from error sequences. However, error sequences that are assigned to the wrong clusters do not affect these measures. When error sequences are not grouped with their source barcodes, the estimated counts of the source barcodes deviate from the true counts. In order to fully assess the accuracy of error sequence assignment, the estimated counts of the barcodes are compared with the true counts in Figure 2b for each method on Dataset B. For a comparison of the estimated counts on Datasets A and C, we refer to Supplementary Figure S3. We see that Shepherd is less prone to overestimation of the barcode counts compared to existing methods. We attribute this difference to the fact that Shepherd considers all nearby putative barcodes before assigning an error sequence to the closest one. Therefore, Shepherd is able to accurately determine which error sequences belong to each putative barcode in cases when two or more putative barcodes are close in the sequence space.

Both Shepherd and Bartender support error correction across multiple time points. We evaluate the multiple time point accuracy of each method using synthetic data of 500 000 barcoded lineages, 5000 of which were given a growth advantage to simulate selection. The details of the simulation procedure for the multiple time points data are given in Supplementary Section S4B. To assess the accuracy of Shepherd and Bartender, we consider the mean absolute error (MAE) at each time point (Fig. 2c). One can see that Shepherd achieves a considerably lower MAE compared to Bartender across all time points. One can also see that while the MAE of Bartender remains relatively constant over time, the MAE of Shepherd decreases with time. This is because false negatives introduced by Shepherd in the first time point are corrected at later time points as described in Section 2.3.

### 3.2 Experimental Illumina HiSeq data

In this section, we compare the methods on the experimental sequencing data (dataset is available from the NIH Sequence Read Archive with accession number SRR5747458) obtained by Levy *et al.* (2015) using the Illumina HiSeq sequencing platform. In short, genetic barcodes were inserted into a clonal population of *S.cerevisiae*, grown in a serial batch culture setup and transferred every eight generations. At each time point, DNA was extracted and sequenced to generate the barcode datasets. Here we consider the dataset generated in the first time point. The dataset consists of 2 450 766 unique sequences and the total read count is ∼136 million. In contrast to the synthetic datasets, the optimal clustering of the barcode reads is unknown in this case. Therefore, we will check for consistency in performance with the synthetic data and employ cluster validation measures to evaluate the clustering quality for each method.

For Shepherd, the log-Bayes Factor is set to its default value –4 and the substring length is set to *k *=* *3. The other parameters of Shepherd are automatically determined based on the input data and are summarized in Supplementary Table S2. For Bartender, we use the same parameter settings used by Zhao *et al.* (2018) on the same dataset. Starcode is applied using its default setting and the distance threshold set to 2.

The number of putative barcodes identified by Shepherd, Bartender and Starcode are 1 034 911, 1 038 600 and 1 131 999, respectively. The number of barcodes identified in common by the methods is summarized in Supplementary Figure S5. We evaluate the clustering of each method using a measure of cluster compactness, termed the effective cluster radius *r_e_*. For a given cluster, *r_e_* is defined as the average Hamming distance between its highest count sequence and all other sequences in the same cluster, with the highest count sequence treated as the cluster center. A small *r_e_* for a cluster implies that the cluster is compact, i.e. sequences in the cluster are close to the cluster center.

**Fig. 3.**
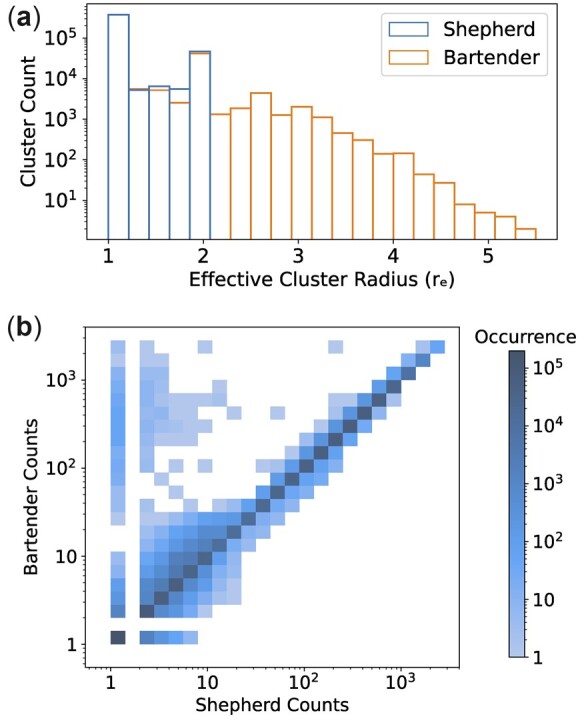
(**a**) Distribution of the effective cluster radius *r_e_* for each method for all clusters containing at least two sequences. There are 439 658 and 446 168 such clusters for Shepherd and Bartender, respectively. (**b**) A 2D histogram of the cluster read counts estimated by Shepherd and Bartender including the barcodes identified by both methods. The colorbar indicates the number of barcodes in each bin

Figure 3a compares the distributions of *r_e_* for Shepherd and Bartender. We refer to Supplementary Figure S4 for a plot comparing all three methods. For Shepherd, *r_e_* is between 1 and 2 for all clusters. For Bartender, most clusters have an effective cluster radius close to 1 but 13 205 clusters (∼3% of clusters) have values of *r_e_* >2, with some clusters having values of *r_e_* >5. Because clusters with a high *r_e_* have low compactness, it is possible that these clusters contain error sequences that originate from different true barcodes. In that case, one would expect Bartender to overestimate the counts of the barcodes to which error sequences are assigned incorrectly. We see from Figure 3b that Bartender tends to estimate higher barcode counts than Shepherd, especially for low count barcodes. This is also consistent with the clustering results on synthetic data (see Fig. 2b).

It should be noted that cluster compactness alone is not sufficient to be a good measure of clustering quality. This is because increasing the cluster count always increases cluster compactness, but eventually leads to over-clustering. Therefore, we must also consider the number of clusters identified by each method to understand why Shepherd produces more compact clusters than Bartender. While the total cluster counts of Shepherd and Bartender are similar, Shepherd identifies 3689 fewer clusters than Bartender. This suggests that the more compact clustering of Shepherd should be attributed to superior clustering quality as opposed to over-clustering.

## 4 Discussion

In summary, Shepherd exploits the pigeonhole principle to efficiently find neighborhoods for each sequence using the *k*-mer indexing system. By utilizing an estimated per nucleotide error rate, Shepherd can accurately classify sequences as either true barcodes or error sequences and is able to reliably assign error sequences to their source barcodes. In terms of both synthetic data and experimental sequencing data, we have demonstrated that Shepherd is significantly more accurate than other state-of-the-art methods for DNA barcode read clustering.

These improvements in error correction accuracy have a number of implications for lineage tracking using DNA barcodes. Fundamentally, these improvements lead to higher resolution lineage tracking, with more accurate estimates of the relative counts of lineages in a population. In particular, Shepherd introduces significantly fewer spurious lineages when compared with previous methods. Notably, Shepherd also introduces the novel capability of tracking lineages that are undetectable in the first time point but emerge at later time points. Consequently, Shepherd enables more reliable estimates of biologically relevant quantities inferred from lineage tracking data, such as the beneficial mutation rate or the number of lineages without a beneficial mutation (Levy *et al.*, 2015).

Recall that a number of simplifying assumptions were made to allow Shepherd to operate using a single per nucleotide error rate estimate. Specifically, Shepherd assumes that errors are equally likely at every position of the barcode and that all nucleotides are equally likely to replace the original one when an error occurs. Since substitution error rates tend to be higher at the end of a sequencing read (Pfeiffer *et al.*, 2018), a promising direction for future work is to explore the possibility of estimating separate error rates for each nucleotide position. Phred quality scores that estimate the sequencing error probabilities at each nucleotide position could conceivably be used for this purpose (Ewing and Green, 1998).

While Shepherd is designed for error correction of DNA barcodes, its applicability extends to any error correction task involving errors in short DNA sequences (e.g. <100 nucleotides). Furthermore, the *k*-mer indexing system presented here enables computationally efficient identification of sequence neighborhoods, and can be applied more broadly to any neighborhood identification task involving the Hamming distance. Such tasks are not limited to genomics and arise also in transcriptomics (Macmanes and Eisen, 2013).

## Supplementary Material

btac395_Supplementary_Data
