# Supplementary material for: Shepherd: accurate clustering for correcting DNA barcode errors
Source: Bioinformatics. 2022 Jun 16;38(15):3710–6. doi: 10.1093/bioinformatics/btac395 (PMC9344852; doi:10.1093/bioinformatics/btac395)
Supplement: btac395_Supplementary_Data [file btac395_supplementary_data.pdf]

Supplementary Materials

1. BAYESIAN HYPOTHESIS TEST

When a given sequence is within Hamming distance ϵ of a putative barcode, it needs to be classified as either an error sequence or a true barcode. This decision should account for both sequences and their counts together with the estimated per nucleotide error rate ρ.

Let S_c denote the sequence under consideration and let f_c denote its read count. Furthermore, let S_p denote the sequence of the neighboring putative barcode with read count f_p. The Hamming distance between the sequences is given by d, such that $d \leq \epsilon$.

Two competing models for the sequence will be considered. In the first model, M_1, the sequence S_c originated from the nearby putative barcode S_p through substitution errors. In the second model, M_2, the sequence S_c is itself a true barcode generated independently of the nearby putative barcode S_p.

The marginal likelihood of each model M_i takes the form,

$$P(f_c, S_c \mid S_p, f_p, \rho, M_i). \tag{S1}$$

Naturally this marginal likelihood will depend greatly on the model we are considering.

1.A. Marginal Likelihood of Model M_1

To find a computable expression for the marginal likelihood of model M_1 the probability chain rule is used to obtain,

$$P(f_c, S_c \mid S_p, f_p, \rho, M_1) = P(f_c \mid S_c, S_p, f_p, \rho, M_1) P(S_c \mid S_p, \rho, M_1), \tag{S2}$$

where we have used $P(S_c \mid S_p, f_p, \rho, M_1) = P(S_c \mid S_p, \rho, M_1)$, i.e. the probability of observing the sequence S_c only depends on the sequence of the nearby putative barcode S_p, but not on its read count f_p. This is because $P(S_c \mid S_p, \rho, M_1)$ is the probability of converting the sequence S_p to S_c in one trial/reading. Consequently, while f_p is directly related to the total number of trials, given by the true count of S_p in the population, it does not affect the probability that S_p is converted to S_c in one of these trials.

Given this interpretation a computable expression for $P(S_c \mid S_p, \rho, M_1)$ can also be found. Each time an error occurs at a nucleotide position, there are 3 nucleotides to replace the correct one. We assume that each one of these 3 possibilities is equally likely. Since the distance between S_p and S_c is given by d it follows that the probability of converting S_p to S_c in one trial is estimated by,

$$P(S_c \mid S_p, \rho, M_1) = \hat{p}_{\text{pc}} = (\rho/3)^d (1-\rho)^{l-d}, \tag{S3}$$

where it is assumed that the error rate is the same at each nucleotide position, and that errors occur independently at each nucleotide position.

We can see that $\hat{p}_{\text{pc}}$ is normalized by summing over all possible sequences S_c to obtain,

$$\begin{aligned} \sum_{S_c} P(S_c \mid S_p, \rho, M_1) &= \sum_{k=0}^{l} \binom{l}{k} 3^k (\rho/3)^k (1-\rho)^{l-k} \\ &= \sum_{k=0}^{l} \binom{l}{k} \rho^k (1-\rho)^{l-k} = 1. \end{aligned} \tag{S4}$$

The term 3^k appears in the first equality since there are 3 possible nucleotides at each of the k positions where the two sequences differ.

An expression for $P(f_c \mid S_c, S_p, f_p, \rho, M_1)$ is also needed to determine the marginal likelihood of model M_1. It is the probability of observing the read count f_c for the sequence S_c given that it originated from the neighboring putative barcode S_p with the observed read count f_p. We assume that S_p has some unobserved true count in the population that we will denote n. We can think about the process of sequencing S_p as n independent Bernoulli trials, each one has a probability $\hat{p}_{\text{pc}}$ (Eq. S3) of converting S_p to S_c. From this point of view f_c follows a binomial distribution

with parameters n and $\hat{p}_{\text{pc}}$. We want to evaluate the probability of observing f_c under this model. To proceed, the unobserved parameter n needs to be estimated.

Consider the distribution of f_p. Since we are assuming that S_p is a true barcode, it follows that f_p is its observed count in the population. In particular, it is the number of times S_p was sequenced without errors. The probability of no error occurring in one reading of S_p is estimated by $\hat{p}_{\text{ne}} = (1-\rho)^l$. Consider each reading as an independent Bernoulli trial with probability $\hat{p}_{\text{ne}}$ of introducing no substitution errors. It follows that f_p is a sample from a binomial distribution with parameters n and $\hat{p}_{\text{ne}}$. Consequently, we can obtain the maximum likelihood estimate of n given by,

$$\hat{n}_{\text{mle}} = \left\lfloor \frac{f_p}{\hat{p}_{\text{ne}}} \right\rfloor. \tag{S5}$$

where $\lfloor \cdot \rfloor$ denotes the floor function. For a more thorough discussion on the estimation of this parameter we refer to Blumenthal and Dahiya (1981). Under the current model, M_1, both sequences originated from the same source barcode and so we need to ensure that our estimate of n is not less than $f_p + f_c$. Therefore, our estimate of n is given by,

$$\hat{n} = \max\left(\hat{n}_{\text{mle}}, f_p + f_c\right). \tag{S6}$$

Using this estimate we obtain the following expression for the desired probability,

$$P(f_c \mid S_c, S_p, f_p, \rho, M_1) = p(f_c; \hat{n}, \hat{p}_{\text{pc}}) = \binom{\hat{n}}{f_c} \hat{p}_{\text{pc}}^{f_c} (1-\hat{p}_{\text{pc}})^{\hat{n}-f_c},$$

where $p(k; n, p)$ denotes the probability mass function of a binomial distribution with parameters n and p evaluated at k. The marginal likelihood of our first model M_1 (Eq. S2) is now estimated by,

$$\begin{aligned} P(f_c, S_c \mid S_p, f_p, \rho, M_1) &= P(f_c \mid S_c, S_p, f_p, \rho, M_1) P(S_c \mid S_p, \rho, M_1) \\ &= p(f_c; \hat{n}, \hat{p}_{\text{pc}}) \hat{p}_{\text{pc}}. \end{aligned} \tag{S7}$$

1.B. Marginal Likelihood of Model M_2

In a similar way we can also find an expression for the marginal likelihood of model M_2. As before the probability chain rule is used to obtain,

$$P(f_c, S_c \mid S_p, f_p, \rho, M_2) = P(f_c \mid \rho, M_2) P(S_c \mid S_p, M_2). \tag{S8}$$

In Eq. (S8), we use the property that the read count of the sequence under consideration, S_c, is independent of the nearby putative barcode S_p and its read count f_p. However, since S_c and S_p are distinct sequences, the probability of observing S_c will not be independent of S_p. Furthermore, the probability of observing S_c does not depend on the error rate ρ, since it is a randomized sequence under model M_2. On the other hand, the probability of observing f_c will depend on ρ. This becomes clear if we think about f_c as the number of times S_c was read without errors.

Since S_c is a random DNA sequence under M_2 with 4 possible nucleotides at each of the l positions it follows that,

$$P(S_c \mid S_p, M_2) = \frac{1}{4^l - 1} \approx \frac{1}{4^l}. \tag{S9}$$

The probability $P(f_c \mid \rho, M_2)$ is more difficult to determine. It is the probability of observing the read count f_c for a true barcode S_c in the population, given the error rate ρ. Since the observed count distribution of the sequences includes error sequences, the distribution of the observed true barcode counts is unknown. What we do know is the maximum observed count f_{max}. Given this maximum, we have a range for the possible count values between 1 and f_{max}. With no additional information we want to assume as little as possible about the count distribution. This is achieved by choosing the maximum entropy distribution, given by the discrete uniform distribution in our case. It follows that for $f_c \in [1, f_{\text{max}}]$,

$$P(f_c \mid \rho, M_2) = \frac{1}{f_{\text{max}}}. \tag{S10}$$

The marginal likelihood of model M_2 is now given by,

$$P(f_c, S_c \mid S_p, f_p, \rho, M_2) = P(f_c \mid \rho, M_2) P(S_c \mid M_2) = \frac{1}{4^l f_{\text{max}}}. \tag{S11}$$

1.C. Sequence Classification using Bayes Factor

The marginal likelihood of each model is now computable. To compare the models and to determine which model describes a given sequence better we will use the log Bayes factor, $\ln K$, the logarithm of the ratio between the marginal likelihoods of model M_1 and M_2 given by,

$$\begin{aligned} \ln K &= \ln P(f_c, S_c \mid S_p, f_p, \rho, M_1) - \ln P(f_c, S_c \mid S_p, f_p, \rho, M_2) \\ &= \ln p(f_c; \hat{n}, \hat{p}_{\text{pc}}) + \ln \hat{p}_{\text{pc}} + l \ln 4 + \ln f_{\text{max}}. \end{aligned} \tag{S12}$$

To make a decision about whether the current sequence is an error sequence or a true barcode we will consider M_1 as our null model. We only want to reject this model if its marginal likelihood is significantly lower than the marginal likelihood of the alternative model M_2. By default, we will reject the null model if $\ln K \leq -4$, i.e if the marginal likelihood of model M_2 is approximately 55 times greater than the marginal likelihood of model M_1. The threshold can be adjusted by the user to control the trade-off between false positives and false negatives. Increasing the threshold will increase the number of false positives while reducing the number of false negatives.

2. MULTIPLE TIME POINT MODE

Instead of performing clustering at each time point independently, Shepherd constructs the k-mer Index at the first time point and only performs clustering for the first time point using the procedure described in section 2.2 in the main text. For subsequent time points, Shepherd treats the error correction task as a classification problem: Let t_n denote the nth time point. Once the clustering is performed for t_1, each sequence at t_2 is assigned to one of the clusters identified at t_1. Let us consider a sequence from t_2 that we want to assign to a cluster from t_1. To find the most suitable cluster for the sequence, the k-mer Index from t_1 is used to find its closest putative barcode from t_1. The sequence is then assigned to the cluster of this putative barcode if it is within distance ϵ. If two or more putative barcodes at t_1 have the same Hamming distance to the sequence under consideration at t_2 and appear in its ϵ-neighborhood, the sequence is assigned with the one in the higher count cluster at t_1. This is because the sequence is more likely to belong to the higher count cluster given that its distance to the putative barcodes is equal. At later time points the same classification procedure is used to assign sequences from time point t_m ($m > 1$) to the putative barcodes from the previous time point t_{m-1}.

The computational advantage of the above multiple time point procedure is that the construction of the k-mer Index is only performed at the first time point, reducing the computational time required for error correction in subsequent time points. Moreover, by assigning sequences from later time points to clusters from previous time points, one connects barcodes from different time points and performs error correction simultaneously.

2.A. Identification of Emerging Barcodes

Some barcodes exist in the population but may not be detectable in the first time point due to low sequencing depth and a low barcode count. However, these barcodes may rise in frequency at later time points and could have a significant impact on the evolutionary dynamics of the population.

Shepherd is capable of identifying barcodes that emerge at later time points. This is done for each time point after the sequences have been classified to putative barcodes from the previous time point using the procedure described in the previous section.

Barcodes that emerge within the ϵ-neighborhoods of putative barcodes are separated from these barcodes. This is done by using the statistical test described in section 1 to determine if a sequence is an error sequence or a new putative barcode. If a new putative barcode is identified all sequences in the same cluster are reassigned to either the original putative barcode or the new putative barcode, using the statistical test to determine which barcode each sequence is more likely to originate from. Furthermore, unassigned sequences are assigned to one of these new putative barcodes if they are within distance ϵ.

Unassigned sequences not found in the ϵ-neighborhood of any putative barcode are clustered using the procedure described in section 2.2 in the main text. This step yields new putative barcodes that are discarded unless they are also found in the next time point. This is done to prevent the formation of false positives. When a new putative barcode is identified its k-mers are added to the k-mer index so that it can be found at later time points.

2.B. Algorithm

The procedure for multiple time point error correction can be summarised in steps. Given a time point t_m ($m > 1$) we perform the following steps to achieve accurate error correction.

1. Classify sequences at time t_m to putative barcodes from time t_{m-1}.
2. Separate emerging barcodes that were assigned to putative barcodes from t_{m-1} using the statistical test.
3. Reclassify all sequences in separated clusters to either the original putative barcode or the new putative barcode using the statistical test.
4. Assign sequences not assigned in step 1 to new putative barcodes introduced in step 3, if they are within distance ϵ.
5. Cluster remaining unassigned sequences using the single time point procedure. New putative barcodes from this step are discarded unless an exact match also appears at time t_{m+1}.

3. PARAMETER SELECTION AND OPTIMIZATION

In this section we detail how the parameters of Shepherd are automatically determined based on the input data. We will also discuss how the clustering procedure is optimized to improve performance. The per nucleotide substitution error rate ρ is determined from the input data using the procedure described in section 2.A. The maximum distance ϵ for merging sequences and the substring length k used for k-mer indexing can also be determined from the input data using the procedures described in sections 2.B and 2.C, respectively. Furthermore, the parameters τ and f_t introduced for performance optimization are also estimated from the input data as described in section 2.D.

It is important to note that the only parameters that impact the clustering results are ϵ and ρ. Specifically, the ϵ parameter dictates which sequences are considered for merging and ρ is used in the statistical test to determine if a sequence is an error sequence or a true barcode. The other parameters of Shepherd only affect computational time and memory usage.

3.A. Determining ρ

Given the input data the parameter ρ is estimated by considering all possible single-nucleotide polymorphisms (SNPs) of the highest count sequences. Specifically, we consider the N_h highest count sequences in the input data. For each of these sequences we find the set of all possible SNPs. Since the highest count sequences in the input data are almost certainly true barcodes the count of these sequences corresponds to the number of times these barcodes were sequenced without errors. Let n_0 denote the combined counts of the N_h highest count sequences and let n_1 denote the combined counts of their SNPs.

Under the binomial model for sequencing errors proposed in the previous section the probability of reading a sequence without errors is given by,

$$P(X = 0) = (1 - \rho)^l. \tag{S13}$$

Under the same model the probability of a SNP during sequencing is given by,

$$P(X = 1) = l\rho(1 - \rho)^{l-1}. \tag{S14}$$

To obtain an estimate of ρ we consider the following ratio of Eq. (S13) and Eq. (S14),

$$\frac{P(X = 1)}{P(X = 0)} = \frac{l\rho(1 - \rho)^{l-1}}{(1 - \rho)^l} = l\frac{p}{1 - p}. \tag{S15}$$

Empirical probability estimates of the probabilities $P(X = 0)$ and $P(X = 1)$ are given by $\hat{P}(X = 0) = n_0/n$ and $\hat{P}(X = 1) = n_1/n$, respectively. Here n is the unknown total number of reads when sequencing the N_h highest count sequences. By substituting these estimates in Eq. (S15) we obtain,

$$\frac{\hat{P}(X = 1)}{\hat{P}(X = 0)} = \frac{n_1}{n_0} = l\frac{p}{1 - p}. \tag{S16}$$

Note that the unknown quantity n cancels out in Eq. (S16) and we obtain a relationship between ρ and the known quantities l, n_0 and n_1. By rearranging the terms in Eq. (S16) we now obtain the following estimate of the error rate ρ,

$$\hat{\rho} = \frac{n_1}{n_1 + l n_0}. \tag{S17}$$

By default the number of sequences used for the estimation is set to $N_h = \min(N, 500)$, where N is the total sequence count.

3.B. Determining ϵ

We will start by fixing ϵ appropriately. We want to choose ϵ so that the vast majority of error sequences are within the ϵ-neighborhoods of their source barcodes. However, we do not want to choose a larger ϵ than necessary. Firstly, the memory and time complexity of the algorithm increase for larger values of ϵ. This is because a larger ϵ necessitates that the sequence is divided into more partitions with smaller k, since we require that the number of partitions $p > \epsilon$. In general, this will increase the number of k-mer combinations and consequently the number of entries in the k-mer index. As a result, the index will take up more space in memory and will take longer to construct. Another reason that we want to avoid choosing a larger ϵ than necessary is that the k-mer neighborhoods become larger as ϵ increases. Since we search the k-mer neighborhoods for putative barcodes this leads to an increase in search time. If the distance between a sequence under consideration S_c and a putative barcode S_p is large enough our statistical test will classify S_c as a putative barcode regardless of the counts of the sequences. Therefore a reasonable choice for ϵ is the largest distance such that S_c could still be classified as an error sequence for some count combinations of f_p and f_c. To find this distance we start by considering count combinations that will clearly favour model M_1 for a given distance d between S_c and S_p. We fix the count of the putative barcode to $f_p = f_{\max}$. It remains to find the value of f_c that maximizes the marginal likelihood of model M_1.

From Eq. (S7) we see that maximizing the marginal likelihood of model M_1, with respect to f_c, is equivalent to finding the mode of the binomial distribution with parameters $\hat{n}$ and $\hat{p}_{\text{pc}}$. However, since $\hat{n}$ is a function of f_c we will replace it with $\hat{n}_{\text{mle}}$ to determine the mode. We can do this since we know that the value of f_c that maximizes the marginal likelihood of model M_1 will be much smaller than $f_p = f_{\max}$, since it represents the most likely count of an error sequence originating from sequence S_p. Consequently, it is safe to assume that $\hat{n}_{\text{mle}} > f_p + f_c$, which implies $\hat{n} = \hat{n}_{\text{mle}}$. Since $f_c > 0$ it follows that given $f_p = f_{\max}$ and a distance d between S_c and S_p, the value of f_c that maximizes the marginal likelihood of model M_1 is given by,

$$f_c = \max\left(\lfloor(\hat{n}_{\text{mle}} + 1)\hat{p}_{\text{pc}}\rfloor, 1\right). \tag{S18}$$

To find ϵ we apply our statistical test using the count combination f_c (as defined in Eq. S18) and $f_p = f_{\max}$, for increasing values of d. The largest value of d for which S_c is classified as an error sequence will be chosen as the value for ϵ.

3.C. Determining k

When choosing k we need to make sure that $p > \epsilon$. However, in most cases there are several choices of k that satisfy this constraint. On the one hand, we want to choose a small k so that $k\epsilon - \epsilon$ is small, this corresponds to the distance between the dashed circle and the solid circle being small in Figure 2. Since we only consider ϵ-neighbors for merging, this ensures that the number of irrelevant sequences in each neighborhood with distance greater than ϵ are minimized. This will decrease the size of each neighborhood resulting in shorter search times. However, as we mentioned previously a smaller k will also increase the number of k-mer combinations, increasing the memory use and running time of the algorithm.

To find a reasonable value for k we will only consider the true barcodes in the absence of errors. The reason for this is that error sequences will be close to their source barcodes in sequence space. Consequently, if we focus on excluding true barcodes, that are not associated with a given true barcode, we are simultaneously excluding many of the error sequences of those distinct barcodes as well. We will also assume that ϵ has already been fixed. It should be noted that the optimal value for k depends on the hardware used for running the algorithm. However, our approach here does not consider the hardware and only attempts to find a reasonable choice based on the theoretical distribution of Hamming distances for random barcodes.

For a given barcode we want to ensure that the number of distinct barcodes in the region between the dashed circle and the solid circle in Figure 2 is small. As discussed in section 2 the Hamming distance from a given barcode to a random barcode follows a binomial distribution with l trials and success probability 3/4. Given this distribution we will require that,

$$P(\epsilon < d \leq k\epsilon) = \sum_{d=\epsilon+1}^{k\epsilon} \binom{l}{d} \left(\frac{3}{4}\right)^{d} \left(\frac{1}{4}\right)^{l-d} < \frac{1}{2}. \tag{S19}$$

This constraint ensures that for a given barcode the majority of distinct barcodes are expected to be either within distance ϵ or beyond distance $k\epsilon$. This ensures that we do not pick a k that is too large. Given Eq. (S19) and the constraint $p > \epsilon$ we will now pick the largest integer that satisfies both as our value for k. The constraint given in Eq. (S19) is somewhat arbitrary since there is no inherent reason to choose 1/2 as our threshold. Nevertheless, we have chosen it here since it resulted in appropriate choices for k in practice. For example, our parameter selection scheme found $k = 4$ to be the optimal value for synthetic datasets A and B (see table S2). For both datasets the barcode length was $l = 20$ and $\epsilon = 3$ was chosen. By considering the other two possible values for k, 2 and 5, we can see why this choice is optimal. If $k = 2$, the k-mer index will take up too much space in memory. If $k = 5$, the index takes up slightly less space in memory compared to $k = 4$. However, this choice increases the k-mer neighborhood size significantly, since $k\epsilon = 5 \times 3 = 15$ for $k = 5$ compared to $k\epsilon = 4 \times 3 = 12$ for $k = 4$. If we consider the hamming distance distribution for true barcodes we see that many of the distinct barcodes will be within hamming distance 12 to 15. Therefore, $k = 5$ is not a suitable choice since it would cause a large number of unrelated barcodes to be included in the k-mer neighborhoods.

3.D. Performance Optimization

The statistical test described in section 1 is important for classifying a sequence in cases when it is unclear whether it is a true barcode or an error sequence. However, a simple threshold for the distance d or the count f_c will suffice to classify the sequence accurately in many of the cases encountered. By introducing appropriate thresholds to identify these cases we can avoid performing the statistical test repeatedly, which leads to a reduction in computational time. There are primarily two common classes of sequences that we want to focus on for performance optimization.

The first common case is that the sequence has a high enough count that regardless of how close it is to a neighboring barcode, it will still be very likely to be a true barcode. For these cases we want to find a high count threshold f_t, such that any sequence with count $f_c \geq f_t$ is more likely to be a true barcode than an error sequence. To do this we consider the case when we have a sequence S_c, with the smallest nonzero Hamming distance $d = 1$ to the true barcode S_p. Furthermore, we consider the case when $f_p = f_{\max}$. The idea is to maximize the marginal likelihood of model M_1. To find f_t we perform our statistical test for increasing values of f_c, starting from the value of f_c given in equation Eq. (S18). We are looking for the smallest value of f_c for which the marginal likelihood of model M_2 is greater than the marginal likelihood of M_1. Consequently, the first value of f_c such that the statistical test classifies the sequence S_c as a true barcode will be chosen as the count threshold f_t. Once we have obtained f_t we can classify all sequences with count $f_c \geq f_t$ as true barcodes, without having to perform the test again for each of these cases.

There is also another case that we want to deal with separately to decrease the running time of our algorithm. Many of the error sequences will have count 1. This is because most errors that originate from the same barcode are unique under reasonable assumptions on the error rate, the barcode length and the barcode count distribution. To save time we want to find a distance threshold, τ, such that any sequence with read count $f_c = 1$ that is within Hamming distance τ to a barcode S_p is more likely to be an error sequence than a true barcode, regardless of the count of S_p. As f_p increases, the likelihood that the current sequence is a true barcode decreases. Because of this we will now consider the case when $f_p = 1$, which is when S_c has the highest likelihood of being a true barcode for a given distance d from S_p. We will now start with distance $d = 1$ and perform our statistical test for increasing values of d. The largest value of d for which S_c is classified as an error sequence will be chosen as the value for τ. Any sequence with count 1 within distance τ of its barcode neighbor can now be classified as an error sequence.

Finally, the computational time can be further reduced when computing the Hamming distance between a sequence and its nearby putative barcode. Since sequences beyond distance ϵ are not

considered, a truncated Hamming distance can be employed. For sequences S_a and S_b of length l separated by Hamming distance d, their truncated Hamming distance is defined as,

$$h_t(S_a, S_b) = \begin{cases} d & \text{if } d \leq \epsilon, \\ l & \text{otherwise.} \end{cases} \tag{S20}$$

The truncated Hamming distance allows us to save time by stopping the computation of the Hamming distance once it has exceeded ϵ.

We note that the optimizations described in this section only affect the computational time of the procedure, and do not affect the clustering results.

4. SYNTHETIC DATA

4.A. Single Time Point Data

The single time point evaluation of Shepherd, Bartender and Starcode is based on 3 synthetic datasets.

The procedure for generating these datasets is as follows. First 500 000 barcodes are created, each one with 20 random nucleotides and 6 constant nucleotides. Then a read count is assigned to each barcode by drawing a sample from the exponential distribution with mean 100 and applying the ceiling function to obtain an integer value.

The read count of each barcode corresponds to the number of times it is sequenced. Each time a barcode is sequenced, we perform a Bernoulli trial at each of its nucleotide positions with the chosen error rate as the probability of success. When one of these trials is successful, an error has occurred and the nucleotide at that position is replaced by one of the other 3 nucleotides with equal probability. Once the errors have been introduced, the synthetic datasets consist of a set of unique sequences and their read counts. If a barcode was destroyed in the error generating process, i.e., if every time it was sequenced an error was introduced, all sequences associated with that barcode were removed from the dataset. This was done to simplify the evaluation, since destroyed barcodes that have been clustered correctly are difficult to distinguish from false positives.

Dataset	A	B	C
ϵ	3	3	4
k	4	4	3
τ	2	2	3
f_t	16	22	35

Table S1. Parameters used for Shepherd on each dataset. ϵ is the maximum Hamming distance considered for merging a given sequence with a putative barcode. The parameter k controls the length of the k-mers. τ is the maximum Hamming distance for which a count 1 sequence is merged with a putative barcode within distance ϵ without performing the statistical test. Finally, f_t is a count threshold and all sequences with count greater than f_t are classified as true barcodes without performing the statistical test.

4.B. Simulation Procedure for Multiple Time Point Data

To generate synthetic multiple time point data we start with a single time point synthetic dataset with 500 000 barcodes of length 26 (20 random nucleotides and 6 constant nucleotides). The count of each barcode is obtained by applying the ceiling function to a sample from the exponential distribution with mean 100.

To simulate selective advantage, 5000 of these barcodes are given an increased growth rate of between 5% and 15%. These barcodes are randomly chosen without replacement, with higher count barcodes having a higher probability of gaining a growth rate increase. Specifically, the probability of a barcode gaining a growth rate increase is given by its proportion in the population.The rationale is that once we start tracking the barcodes at the first time point, some lineages may have already acquired a selective advantage previously. As a result, these lineages

are more likely to have a high count in the first time point. Let g_i denote the growth advantage of lineage i. Note that $g_i = 0$ if lineage i is not one of the 5000 lineages with a selective advantage. The growth advantage for a lineage i chosen to receive a selective advantage is given by,

$$g_i = \min(x, 0.15),$$

where x is a sample from the exponential distribution with mean 0.05.

Let $n_{i,t}$ denote the number of individuals/cells of lineage i in generation t and let $p_{i,t}$ denote the probability that a cell in lineage i undergoes mitosis (cell division) once until the next time point, $t+1$. Given that lineage i has $n_{i,t}$ cells at time t we want to simulate the number of cells in the lineage at the next time point, $n_{i,t+1}$. We consider two possible outcomes for each cell in lineage i, either the cell undergoes mitosis once until time $t+1$ with probability $p_{i,t}$, or it dies with probability $1 - p_{i,t}$, independently of all other cells in the population. It follows that $n_{i,t+1}$ has a binomial distribution with $2n_{i,t}$ trials and trial probability $p_{i,t}$. While $n_{i,t}$ is known at time t the cell division probability $p_{i,t}$ must be determined to sample from this binomial distribution and obtain an updated cell count for lineage i.

To determine $p_{i,t}$ while incorporating the growth advantage g_i of lineage i we impose the following constraint,

$$E[n_{i,t+1}] = 2n_{i,t}p_{i,t} = \frac{n_{i,t}(1+g_i)}{\sum_{j=1}^{K} n_{j,t}(1+g_i)} N, \tag{S21}$$

where K is the number of lineages and N denotes the total number of cells in the population in the first time point. Given that g_i has been fixed for each lineage i the only unknown quantity in equation (S21) is $p_{i,t}$ at time t. By solving for $p_{i,t}$ in equation (S21) we obtain,

$$p_{i,t} = \frac{1+g_i}{2\sum_{j=1}^{K} n_{j,t}(1+g_i)} N. \tag{S22}$$

At each time point t the cell division probability for each lineage i is found using equation (S22) and the new cell count for the lineage is obtained by sampling from a binomial distribution with $2n_{i,t}$ trials and success probability $p_{i,t}$.

5. EXPERIMENTAL ILLUMINA HISEQ DATA

5.A. Preprocessing of Experimental Illumina HiSeq Data

Before applying the error correction methods to the experimental data the dataset is filtered to remove extremely low quality sequences. These error sequences have a high enough average per nucleotide error rate that no error correction method is able to reliably correct for them. In fact, in some cases these sequences can accumulate errors at almost all nucleotide positions due to phasing effects (Pfeiffer *et al.*, 2018).

We apply the same sequence quality filter as the one used by Levy *et al.* (2015). In particular, any sequence with an average Phred quality score less than 30 is excluded. Furthermore, we filter out any sequences that do not match the following regular expression:

```
\D*?(.ACC|T.CC|TA.C|TAC.)\D{4,7}?AA\D{4,7}?AA\D{4,7}?TT\D{4,7}?(.TAA|A.AA|AT.A|ATA.)\D*\
```

After the filtering the random barcode regions of correct length 20 are extracted and counted to obtain the final dataset.

5.B. Comparison to Starcode on Experimental Illumina HiSeq Data

We applied Starcode to the experimental Illumina HiSeq data using the default settings of the method, with the distance threshold set to 2. Starcode identified 1 131 999 barcodes, 1 012 458 of these were also identified by Shepherd (see Figure S5). All three methods, Shepherd, Bartender and Starcode identified 963 112 barcodes in common. Figure S4 shows a comparison of the effective cluster radius (r_e) for each method. We see that Starcode has a large number of clusters for which r_e is extremely high. This is a clear sign that Starcode is merging unrelated sequences.

5.C. Runtime Performance

We benchmarked the time performance of Shepherd and Bartender on the experimental sequencing data. Shepherd clustered the data in 9 minutes and 4 seconds and Bartender performed the

clustering task in 27 minutes and 23 seconds. The time measured is the wall-clock time. The benchmark was performed on a desktop PC with an Intel Core I7 6700k processor and 32GB of system memory. The number of threads used by Bartender was set to 4 to match the core count of the processor. We observed that the time performance of the methods varies considerably between the datasets in the study. Specifically, Bartender is faster than Shepherd on all synthetic datasets and Shepherd is faster on the experimental sequencing data.

6. APPENDIX

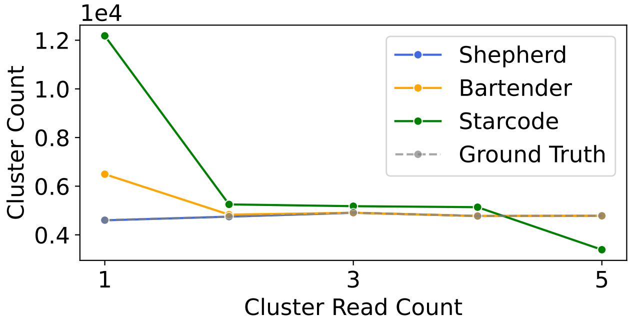

Fig. S1. The number of clusters with low read counts (< 6) for each method compared to the ground truth on dataset A.

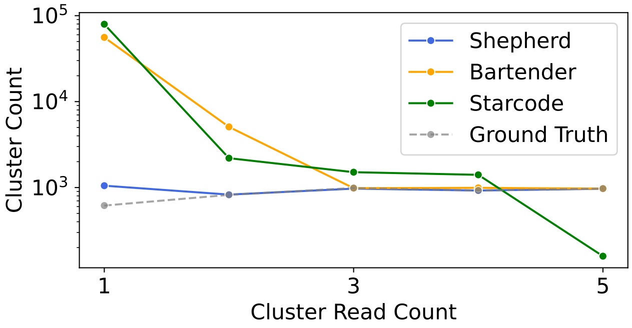

Fig. S2. The number of clusters with low read counts (< 6) for each method compared to the ground truth on dataset C.

Dataset	Illumina HiSeq Data
ρ	0.00055
ϵ	3
k	3
τ	2
f_t	83

Table S2. Parameters used for Experimental Illumina HiSeq Data.

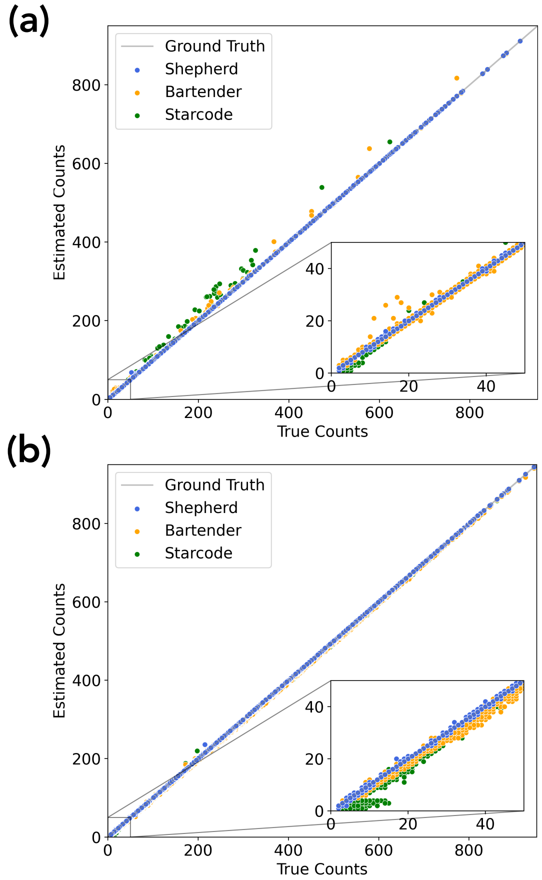

Fig. S3. (a) Estimated barcode counts compared to the true counts for each method on (a) dataset A and (b) dataset C. The figures only include true barcodes that were identified by all three methods. True barcodes for which all three methods estimated the same counts are excluded to emphasize differences in the estimated counts.

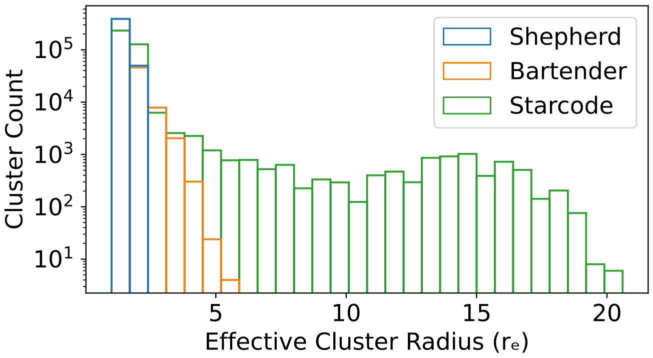

Fig. S4. Distribution of the effective cluster radius r_e on the experimental sequencing data for each method, including all clusters containing at least 2 sequences. There are 439 658, 446 168 and 382 360 such clusters for Shepherd, Bartender and Starcode, respectively.

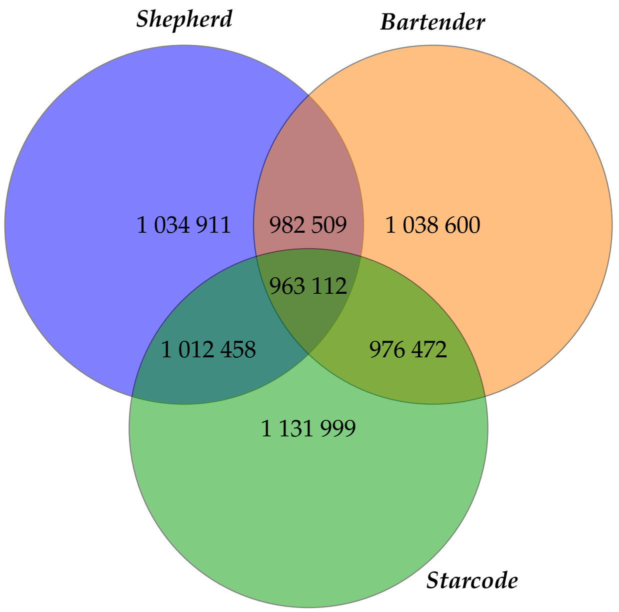

Fig. S5. A Venn diagram showing the number of barcodes identified and shared by each method on the experimental sequencing data.

Algorithm S1. Read Clustering Procedure

Input: sequences (including their read counts), k-mer index, ϵ, error rate estimate ρ
Output: clustered sequences

```
true_barcode_set = ∅
sorted_sequences = sort_by_count(sequences, order=descending)
for seq in sorted_sequences do
    neighborhood = getNeighbors(seq, k-mer index)
    true_barcode_neighbors = true_barcode_set ∩ neighborhood
    if true_barcode_neighbors ≠ ∅ then
        closest_true_barcode = getClosest(seq, true_barcode_neighbors)
        if h(seq, closest_true_barcode) ≤ ϵ then
            class = hypothesis_test(seq, closest_true_barcode, ρ)
            if class == error_sequence then:
                cluster seq with closest_true_barcode
                continue to next iteration
    add seq to true_barcode_set
```

REFERENCES

Blumenthal, S. and Dahiya, R.C. (1981) Estimating the Binomial Parameter n, *Journal of the American Statistical Association*, **76**, 903-909.

Levy, S.F. *et al*. (2015) Quantitative evolutionary dynamics using high-resolution lineage tracking, *Nature*, **519**, 181-186.

Pfeiffer, F. *et al*. (2018) Systematic evaluation of error rates and causes in short samples in next-generation sequencing, *Scientific Reports*, **8**, 10950.
